# The role of knowledge transfer in health policymaking: the US experience

**DOI:** 10.1186/s13584-016-0091-6

**Published:** 2016-06-29

**Authors:** Sherry Glied

**Affiliations:** Robert F. Wagner Graduate School of Public Service, New York University, 295 Lafayette St., 2nd Floor, New York, NY 10012 USA

## Abstract

As Ellen et al. point out, there is room for improvement in knowledge transfer between academic research and policymaking. At the same time, retrospective analyses of health policy often identify research influences on policymaking. Part of this paradox can be explained by the difference between the nature of research and the nature of policymaking. Research necessarily focuses on the past, examining changes that have already taken place. Policymakers want to understand how policy will shape the future. A key element of successful knowledge transfer is the use of mechanisms that allow past research to be used to forecast future policy consequences. One such mechanism is the formal microsimulation model, which translates research-based parameters into out-of-sample forecasts. A more straightforward mechanism is the embedded researcher, who extrapolates from a body of research knowledge to make a policy forecast. These types of mechanisms can supplement formal processes of knowledge transfer.

## Background

Academics and health policymakers everywhere have a tangled relationship. From one angle, the relationship is dysfunctional. Academics around the world complain that their work doesn’t receive the attention it deserves. Policymakers in all countries grumble that academic research rarely provides the right information at the right time to address the pressing questions they face. In response to this disconnect, funders work to encourage collaboration, by rewarding researchers who take extra steps and translate their analyses to make them more easily digestible by policymakers. Recently, the process of translating research into policy has become a subject of research in itself, as exemplified by the recent IJHPR article by Ellen et al. [1].

From a second angle, the relationship between research and policy seems almost too robust. As John Maynard Keynes pointed out back in 1936, “Practical men, who believe themselves to be quite exempt from any intellectual influences, are usually slaves of some defunct economist.” Closer to home, David Chinitz and Victor Rodwin complain about the outsize role of (academic) economic thinking in health policy and management today [2]. In a recent article assessing the roles of public policy and research in the design of the U.S. Affordable Care Act, Erin Miller and I argue that “economic research and thinking have had a growing …impact on health policy…[and] especially … in the design of the Affordable Care Act (ACA), where the influence of academic economic research is broadly and blatantly evident” [3].

As with any such dilemma: “he’s right and he’s right.” Some important and valuable classes of policy research do not translate effectively and in a timely fashion into the practical policy arena. Some policy questions, by their very nature, are not satisfactorily answered by policy researchers. At the same time, policy is profoundly shaped by research and by researchers. What is the source of this paradox? The process in place to aid knowledge transfer, the focus of the article by Ellen et al., is one reason. But the main reason is the nature of the research itself. A better understanding of the features of research that make it more (or less) useful in policymaking can help improve the work of knowledge transfer. In particular, a recognition of the features of research that make it most useful points to the role of academic researchers who play a part within the policy process as vital to the work of knowledge transfer.

## Context

Ellen et al. survey policymakers in Israel to identify impediments to the use of research knowledge in policymaking. The key problems identified are similar to those seen in other countries where knowledge transfer has been studied. These include problems of timing – appropriate research is not available at the moment a decision must be made; accessibility – policymakers cannot find the necessary research in readily digestible form; and relevance – research focuses on the past or on other contexts, making it less useful for policymakers making decisions in Israel today.

Although knowledge transfer activities can help mitigate these challenges, they can only go so far. Most of these problems are inherent in the production of research knowledge in health policy. Health policy research is a hybrid field: it brings together epidemiology and clinical research with social science research, primarily drawn from the disciplines of economics, political science, and sociology. Health policy is also a hybrid in its institutional setting. Some health policy researchers are academics, but some work in think tanks and research organizations. These institutions, in turn, shape the goals of researchers. Health policy researchers in academia are rewarded for publishing novel, interesting articles in academic journals. Timeliness runs a distant second to the demands of rigorous peer review and the desire to generate results that reach beyond a specific problem. Researchers in think tanks and research organizations try to be more responsive to the immediate demands of policymakers. Even so, they typically produce publications for public circulation. That necessitates a pace and a level of review that may frustrate policymakers.

But the institutional imperatives to publish are often the least of the challenges in bringing research into the public space. The conventions of research itself also limit its direct usefulness. Health policy research follows two basic models. One research model, drawing on epidemiology, is primarily descriptive. It often involves the design and deployment of surveys, the design and testing of measures, and fairly straightforward estimates of how often something happens or how many people are affected. Because these processes take a long time, descriptive survey research is most useful to policymakers when it either describes phenomena that are quite stable over time or when the surveys are frequently and consistently repeated, so that changes over time can be consistently measured. Policymakers often deploy this kind of research to provide a justification or impetus for policy changes. The relationship between a single publication describing a survey result and a subsequent policy change can be hard to trace. Yet if we had not known how many uninsured people there were in the United States, what their characteristics were, and how the trends were changing over time, it is very hard to imagine that policymakers would have focused on the problem of insurance coverage.

Survey research of this repeated type is rarely undertaken in academia, though it is occasionally produced by policy research institutes. Instead, most academic health policy research draws more heavily on social sciences and makes use of variation to test hypotheses. A common form of research of this type is an evaluation study, which assesses the consequences of a policy or program change. Without further modification, studies of this type – however rigorous – are rarely directly relevant to national policymakers. The problem is simple: an evaluation study generally examines something that has already happened, but a policymaker is contemplating a novel change. By definition, the policy that is being considered does not already exist. In rare circumstances, a very similar policy change has been tried in a subnational region or in a very similar health system abroad. In a small country like Israel, with a fairly distinctive health system, such comparisons are hard to find. Of course, when the opportunity to draw on very similar experience arises, it can be extraordinarily powerful in the policy process. In the US, the results of analyses of the effects of the Massachusetts health reform were very influential in the design of the Affordable Care Act. But such cases are understandably rare.

Policymakers find most useful those approaches to health policy research that assess the likely effects of realistic and untried policy alternatives and that don’t depend on the rare serendipity of a prior parallel case. These approaches need to be able to make use of prior data and evaluation study results (though these may not be, themselves, directly applicable to an immediate policy problem) to forecast the effects of potential future changes that have not yet occurred. They require drawing out-of-sample inferences from research on the past.

This kind of inference is routine in some parts of policymaking. Budget analysts, who must project the likely costs of a new initiative, draw inferences about the future from observations of the past. A formal analogue in the health policy research field is the microsimulation model. In the US, the Congressional Budget Office, the forecasting arm of the Congress, has a set of simulation models that help lawmakers understanding the consequences and costs of policy proposals they are contemplating. From the perspective of researchers, these models offer an exceptional opportunity to make research findings useful for policy. Estimates of behavioral parameters that can be incorporated into forecast models have been a staple of economics research. Policymakers never read economic research – but model-builders do – and they use the results to fit parameters of their models. Later, sometimes years later, policymakers who seek to understand a potential policy change are unwitting consumers of that research, in the form of model results that build on these findings. In our analysis of the impact of economics on the design of the Affordable Care Act, we found that the Congressional Budget Office and its microsimulation models played a critical role in translating research into policy.

Microsimulation models are complex software programs, often built over many years. In this context, their key feature is that they translate research findings based on prior experiences into forecasts of the future. It takes a considerable effort to build and maintain one or more microsimulation models, and to continually adapt existing models to new policy problems. But there is a less costly, much simpler and more flexible human analogue to the microsimulation model – experienced health policy academic researchers who are embedded in the policymaking process.

Senior health policy researchers who spend time embedded in policy settings bring with them “intellectual influences” born of their own and others’ past research. Like microsimulation models, they use those influences (for better or for worse) to draw inferences about future policies. Senior researchers use their understanding of existing research to make forecasts about the effects of newly-contemplated policy options. This applied policy work itself doesn’t appear in published research -- most often it isn’t published at all -- but it is very much evidence-based.

In the US, this model has been implemented in the executive branch by administrations from both parties. Lead health policy-advising agencies often include academics on leave from their home institutions (as was the case for the author). A number of private foundations have also funded fellowship programs that embed policy researchers in the legislative branch. In all these settings, embedded academics perform a particularly important type of knowledge transfer, drawing inferences from research that would elude policymakers armed only with issue briefs and syntheses. Rather than focusing only on translating research into forms digestible by policymakers, knowledge transfer might well focus on translating the researchers themselves into settings where they routinely interact with policymakers.

## Conclusions

Health policy research has much to bring to the policy process – but the path from research to policy isn’t straightforward. Research looks backward, at what has already happened – policy looks forward, at what might. Forecasting and out-of-sample inference are the tools demanded by policymakers, but these are often unfamiliar and sometimes unpalatable to health policy researchers.

Knowledge transfer can help bridge the divide, but, I believe, equally useful are mechanisms that can translate past policy research into the analysis of potential policies. One such mechanism is the microsimulation model, a formal construct that is populated using results of descriptive surveys and parameter estimates from prior evaluation studies. Well-designed microsimulation models evolve constantly, incorporating new research results as these become available. Microsimulation models allow research about the past to provide quantitative predictions about the future. Another very different mechanism that accomplishes a similar task is an embedded academic. The academic, like a formal model, assimilates prior research and descriptive statistics, and then offers evidence-based predictions of the likely effects of a future policy. In doing so, the academic offers a uniquely powerful mechanism of knowledge transfer.
